# Assessment of Microvessel Permeability in Murine Atherosclerotic Vein Grafts Using Two-Photon Intravital Microscopy

**DOI:** 10.3390/ijms21239244

**Published:** 2020-12-03

**Authors:** Fabiana Baganha, Laila Ritsma, Paul H. A. Quax, Margreet R. de Vries

**Affiliations:** 1 Department of Surgery, Leiden University Medical Center, 2333 ZA Leiden, The Netherlands; F.Baganha_Carreiras@lumc.nl (F.B.); P.H.A.Quax@lumc.nl (P.H.A.Q.); 2 Einthoven Laboratory for Experimental Vascular Medicine, Leiden University Medical Center, 2333 ZA Leiden, The Netherlands; 3 Department of Cell and Chemical Biology, Leiden University Medical Center, 2333 ZC Leiden, The Netherlands; L.M.A.Ritsma@lumc.nl

**Keywords:** angiogenesis, vessel maturity, vessel permeability, hemorrhage, atherosclerosis, two-photon intravital microscopy

## Abstract

Plaque angiogenesis and plaque hemorrhage are major players in the destabilization and rupture of atherosclerotic lesions. As these are dynamic processes, imaging of plaque angiogenesis, especially the integrity or leakiness of angiogenic vessels, can be an extremely useful tool in the studies on atherosclerosis pathophysiology. Visualizing plaque microvessels in 3D would enable us to study the architecture and permeability of adventitial and intimal plaque microvessels in advanced atherosclerotic lesions. We hypothesized that a comparison of the vascular permeability between healthy continuous and fenestrated as well as diseased leaky microvessels, would allow us to evaluate plaque microvessel leakiness. We developed and validated a two photon intravital microscopy (2P-IVM) method to assess the leakiness of plaque microvessels in murine atherosclerosis-prone ApoE3*Leiden vein grafts based on the quantification of fluorescent-dextrans extravasation in real-time. We describe a novel 2P-IVM set up to study vessels in the neck region of living mice. We show that microvessels in vein graft lesions are in their pathological state more permeable in comparison with healthy continuous and fenestrated microvessels. This 2P-IVM method is a promising approach to assess plaque angiogenesis and leakiness. Moreover, this method is an important advancement to validate therapeutic angiogenic interventions in preclinical atherosclerosis models.

## 1. Introduction

Plaque angiogenesis and plaque hemorrhage are major players in the destabilization and rupture of atherosclerotic lesions [1]. Plaque microvessels increase in numbers via angiogenesis during vulnerable stages of the disease, and microvessels density has been associated with the onset of rupture and clinical manifestations [2,3]. Plaque angiogenesis arises from reduced oxygen availability in the plaque, caused by lesion growth and presence of metabolic active inflammatory cells. Triggered by hypoxia, endothelial cells proliferate and migrate from the vasa vasorum to form microvessels to overcome the oxygen demand in the lesion [1]. However, these plaque microvessels are characterized by poor pericyte coverage, lack of cell junctions, and are highly susceptible to leakage of erythrocytes, leucocytes, and plasma lipids, together described as intraplaque hemorrhage [4,5].

Healthy microvessels are present in most organs and tissues, have a well-organized architecture, act as a protection barrier, and provide nutrients and oxygen by passive diffusion to their surroundings. The type of endothelial lining determines the permeability of the vessel. While in continuous microvessels (abundantly found in the ear skin) the endothelial cell lining is uninterrupted, fenestrated microvessels (found in secreting glands) have more pores to increase molecular diffusion of molecules (up to 66 kDa) without compromising their barrier function [6,7]. Contrarily, microvessels in atherosclerotic lesions in their pathological and immature state are thought to have a compromised barrier function and are as such more permeable [4,8,9,10,11].

To date, clinical available imaging techniques to study plaque angiogenesis and subsequent intraplaque hemorrhage, such as PET [12], CT [13], and MRI [14], do not have sufficient spatial resolution to visualize cellular events or image the detailed microvessels network in small size animal models. Two-photon intravital microscopy (2P-IVM), due to its high resolution, can overcome these limitations, allowing detailed 3D reconstructions of plaque angiogenesis and real-time evaluation of target dynamic processes, such as in vivo hemorrhage [7,15,16].

We hypothesized that permeability of healthy continuous and fenestrated microvessels in living mice can be compared to plaque microvessel permeability to assess pathological leakiness. Permeability can be evaluated by quantification of extravasation of 40 kDa-size dextrans [7,17]. It should leak from all vessels [7,17], but is expected to leak more from fenestrated vessels than continuous, and more from pathological vessels than healthy vessels. Moreover, it, is known that capillaries with poor pericytes coverage in context of inflammation can be permeable to large dextrans up to 2000 kDa [17]. Therefore, we also hypothesized that evaluation of 2000 kDa dextran extravasation in the same experimental setting, might be relevant to further assess the pathological permeability.

**Figure 1:**
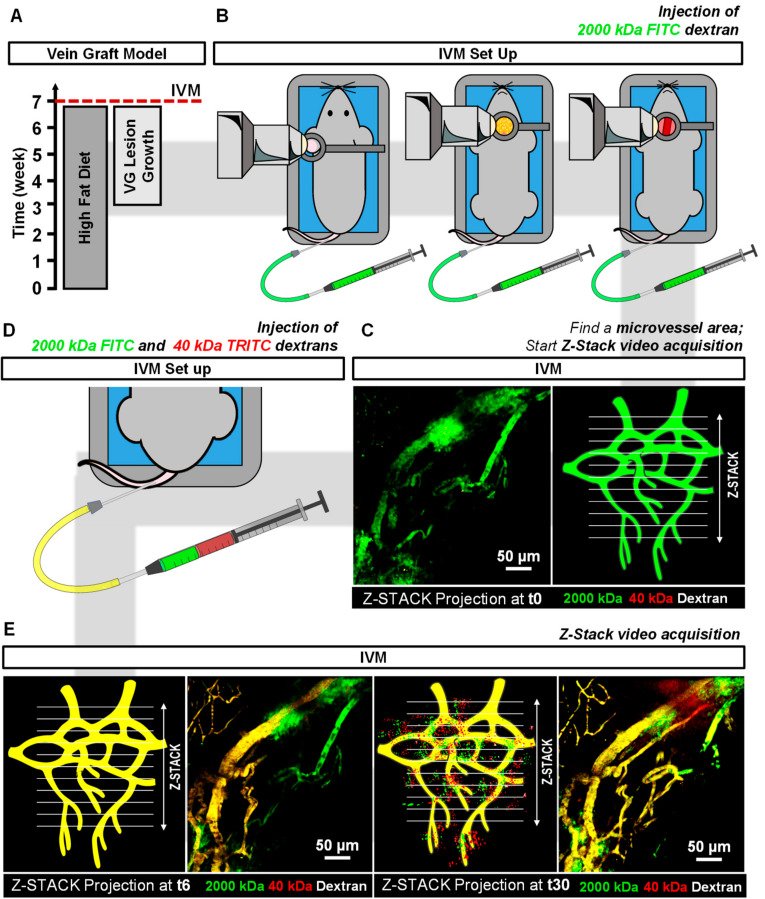
2P-IVM pipeline to image plaque angiogenesis in advance atherosclerotic lesions and to assess dextrans extravasation in continuous, fenestrated and plaque microvessels. (**A**) After 3 weeks of hypercholesteremic diet, ApoE3*Leiden mice (*n* = 12) undergo VG surgery. Four weeks later, (**B**) mice are prepared for IVM and plaque microvessels in the VG lesions, continuous microvessels in ear skin, fenestrated microvessels in parotid glands are imaged by injection of 2000 kDa-FITC dextran intravenously. (**C**) Areas of interest are evaluated by time-lapse Z-stack acquisition over 20 min. (**D**,**E**) Injection of 40 kDa-TRITC 2000 kDa-FITC dextran solution is used to assess vessel permeability by quantification of FICT and TRITC fluorescence extravascular intensities in Z-stack projections.

In this study, we set up an advanced 2P-IVM method to visualize atherosclerotic vein graft (VG) lesions in the neck region of living anesthetized mice. (Figure 1B). With this technique the architecture of adventitial and intimal plaque microvessels in advanced atherosclerotic VG lesions can be visualized (Figure 1C). This model was chosen since in mice, vasa vasorum angiogenesis only occurs at a very old age and most spontaneous atherosclerotic lesions in mice do not show intraplaque angiogenesis [11]. We previously showed that hypercholesterolemic ApoE3*Leiden VG lesions, due to their lesion size and state of hypoxia, present with vasa vasorum derived neovascularization, [8] with the unique characteristic of leaky intimal microvessels and intraplaque hemorrhage [9]. Furthermore, we report a 2P-IVM method to assess vessel permeability by quantification of 40 kDa and 2000 kDa-labeled dextrans extravasation in healthy microvessels and plaque microvessels in real-time (Figure 1D,E). We show that plaque microvessels are pathological more permeable in comparison with healthy continuous and fenestrated microvessels, which advocates for their destabilizing role in plaque rupture.

## 2. Results

### 2.1. Detection of Plaque Angiogenesis in ApoE3*Leiden Mice Vein Graft Lesions

Plaque angiogenesis was detected by 2P-IVM in advanced atherosclerotic lesions of the ApoE3*Leiden mice VG model by injection of the plasma tracer 2000 kDa-FITC dextran as depicted in Figure 1B,C. Here, we imaged for the first time the VG in the neck using high resolution intravital microscopy. We designed a circular metal frame was designed with a coverslip on top connected to a pole by a stalk (Figure 1B,C). The frame could be fixed to the pole at varying heights by a screw. The thin stalk enabled us to image the vessels at high resolution without putting too much pressure on the sternum/chest region or throat.

**Figure 2:**
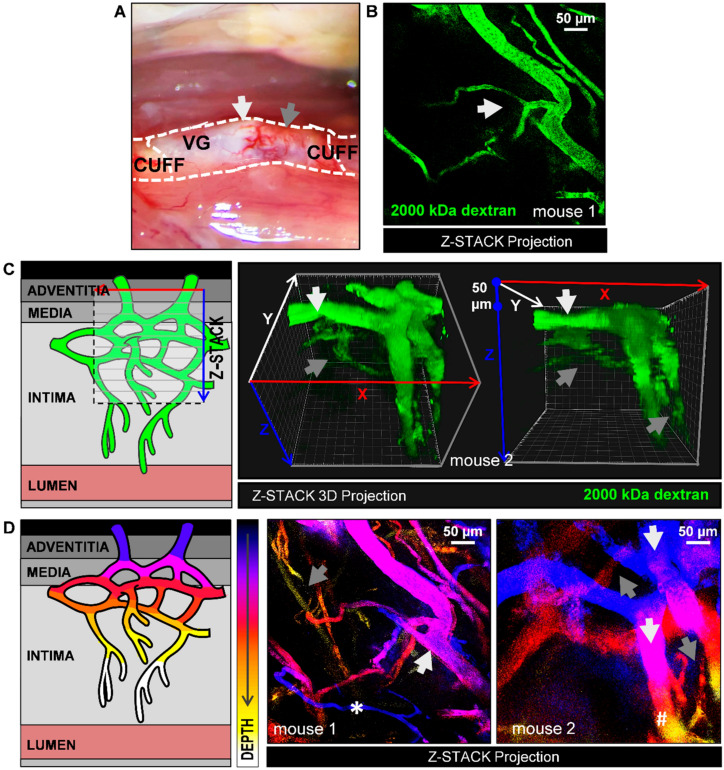
Detection of plaque angiogenesis by 2P-IVM by injection of the plasma tracer 2000 kDa-FITC dextran in atherosclerotic lesions in ApoE3*Leiden mice VGs. (**A**) 28 days after surgery, areas of interest (white arrows: white thick regions, grey arrows: red thin regions) were imaged to study the architecture of plaque microvessels. (**B**) Z-stack projection of plaque microvessels (white arrows: branching). See also Video 1 (total Z-depth of 111 µm, 2 µm step); (**C**) Illustration of Z-stack acquisition in VG lesions and representative Z-stack 3D projections of adventitial (white arrows) and intimal microvessels (grey arrows) from one mouse at two different angles. See also video 2 for 360 view. Note that the large adventitial microvessels are also extending in Z because they are curved around the vein. (**D**) Z-stack projections from two mice with a Z-depth color code filter (white arrows: Adventitial microvessels; grey arrows: Intimal microvessels). Due to the curved nature of the vein: (*) Intimal microvessel is discriminated from adventitial microvessel based on its size, despite being located in a top Z layer; (#) Adventitial microvessel is discriminated from an intimal microvessel based on its size, despite being located in a deeper Z layer.

Thick white fatty atherosclerotic regions were observed along the conduit (Figure 2A). Compared to red thin lesion regions, these white regions show a prominent presence of microvessels. Therefore, white fatty thick spots were chosen to image the architecture of plaque angiogenesis (Figure 2B). FITC signal was homogenously distributed within the intravascular space of the microvessels with no signs of blood flow obstructions. Branching features, characteristic of immature or angiogenic vessels [18] were also observed (Figure 2B and Video 1). In video 1, a 111-µm deep z-stack projection of the image of Figure 2B is shown. This video offers an in depth view of the plaque microvessels throughout the adventitia, media, and intima.

Using 3D stack projections, vasa vasorum angiogenesis is observed throughout the lesion depth (Figure 2C and Video 2). Vasa vasorum angiogenesis expands towards hypoxic plaque areas, (intima) and these intimal microvessels were observed to be narrower than most preexisting adventitial vessels. Bigger adventitial microvessels were usually detected at 50–100 µm depth and narrower intimal microvessel structures, from >100 µm. Accordingly, size and depth were required to discriminate adventitial microvessels from intimal microvessels.

To easily visualize this in a single image, Z depth color coding was used (Figure 2D). As an example of the different variations seen in mice, two examples are given (mouse 1 and mouse 2). Here, vessels which are located deep in Z (in the intimal layer) are differently colored from vessels located high in Z (adventitial layer). Adventitial microvessels are shown in a blue to purple color gradient (Figure 2D, grey arrows) while intimal microvessels are shown in a red to yellow color gradient (Figure 2D, white arrows).

Due to the curved nature of the vein graft, some microvessels not match the color-code (* and #). Indeed, a thin vessel (*) is excluded as an adventitial microvessel based on its caliber, despite being located in a top Z layer. In addition, a thick microvessel (#) is partially located in the deeper Z layers. As can be appreciated in the 3D rendering (Figure 2C), the thick vessel is curved around the vein graft, and therefore extending into the deeper Z layer, and should be excluded as an intimal microvessel. As shown by the represented examples, vessel depth, microvessel caliber, and 3D rendering have to be considered to distinguish adventitial and intimal microvessels.

### 2.2. Evaluation of Dextran in Microvessels

**Figure 3:**
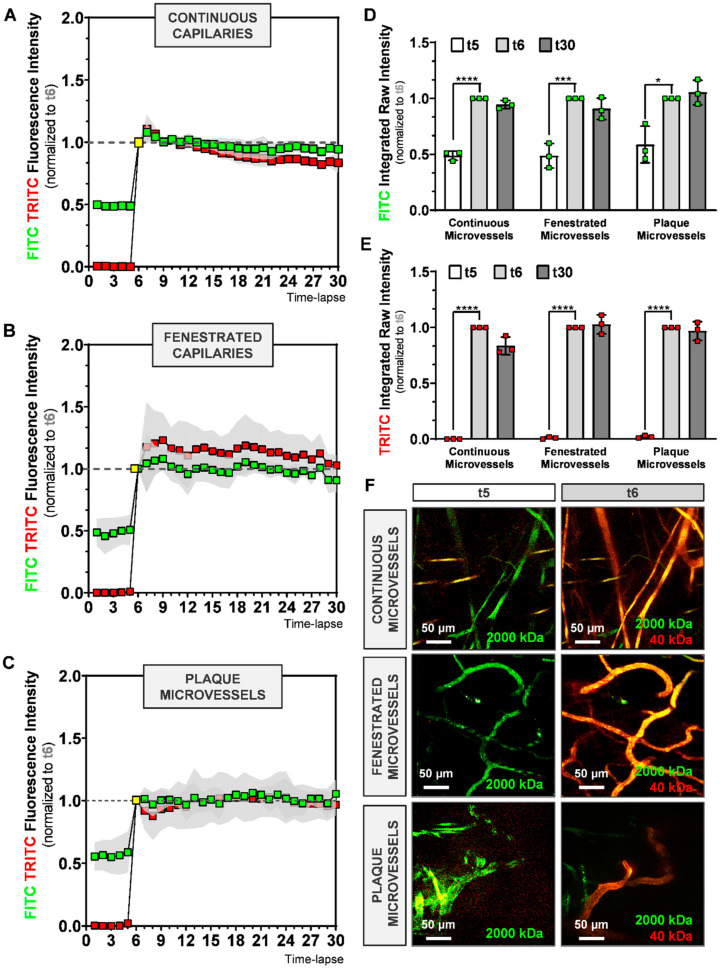
Evaluation of time-lapse imaging of 2000 kDa-FITC and 40 kDa-TRITC RFI inside the microvessel structures of (**A**) continuous, (**B**) fenestrated and (**C**) plaque microvessels. (**D**,**E**) Quantification of FITC and TRITC intravascular RFI at time-lapse 5, 6 and 30. (**F**) Representative max-projection of FITC and TRITC signal in continuous, fenestrated and plaque microvessels at t5 and t6. Data presented as mean ± SD. * *p* ≤ 0.05, *** *p* ≤ 0.001, **** *p* ≤ 0.0001 by 1-way-ANOVA.

To determine the amplitude and speed at which dextrans become visible in the various microvessels, we first injected mice with 2000 kDa-FITC to detect the microvessels and assess a region of interest, and then injected mice with a mixture of 2000 kDa-FITC and 40 kDa-TRITC dextran during timelapse imaging. We plotted the relative fluorescence intensity (RFI, normalized to t6) of 2000 kDa-FITC and 40 kDa-TRITC dextran over time for continuous (Figure 3A), fenestrated (Figure 3B), and plaque microvessels (Figure 3C).

Residual 2000 kDa-FITC fluorescence from the first injection (*t* ≤ 5) was observed in all groups (Figure 3A–C). As shown in Figure 2D, residual FITC RFI at t5 is similar between the groups (Figure 3D).

As expected, after injection of 2000 kDa-FICT and 40 kDa-TRITC dextrans at t5, intravascular FITC RFI increases significantly until t6 in all groups, as demonstrated in Figure 3D (continuous microvessels: *p* < 0.0001, fenestrated microvessels: *p* = 0.0007, plaque microvessels: *p* = 0.0106). TRITC RFI increased even more than FITC RFI, as depicted in Figure 3E, most likely due to the residual FITC from the first injection (continuous microvessels: *p* < 0.0001, fenestrated microvessels: *p* < 0.0001, plaque microvessels: *p* < 0.0001).

During the next 24 image recordings, intravascular FITC and TRITC signal remained stable (~1) in all groups (Figure 3A–C). As shown in Figure 3D,E, FITC and TRITC RFI between t6 and t30 did not differ between continuous, fenestrated and plaque microvessels. Representative sequences of FITC and TRITC fluorescence intensities in continuous, fenestrated and plaque microvessels, observed in real-time, at t5 and t6, are shown in Figure 3F.

Thus, tail vein-injected dextrans appear quickly and with the same dynamics in the various microvessels, and remain so in similar concentration throughout the course of imaging.

### 2.3. Evaluation of Dextran Extravasation into the Extravascular Space

**Figure 4:**
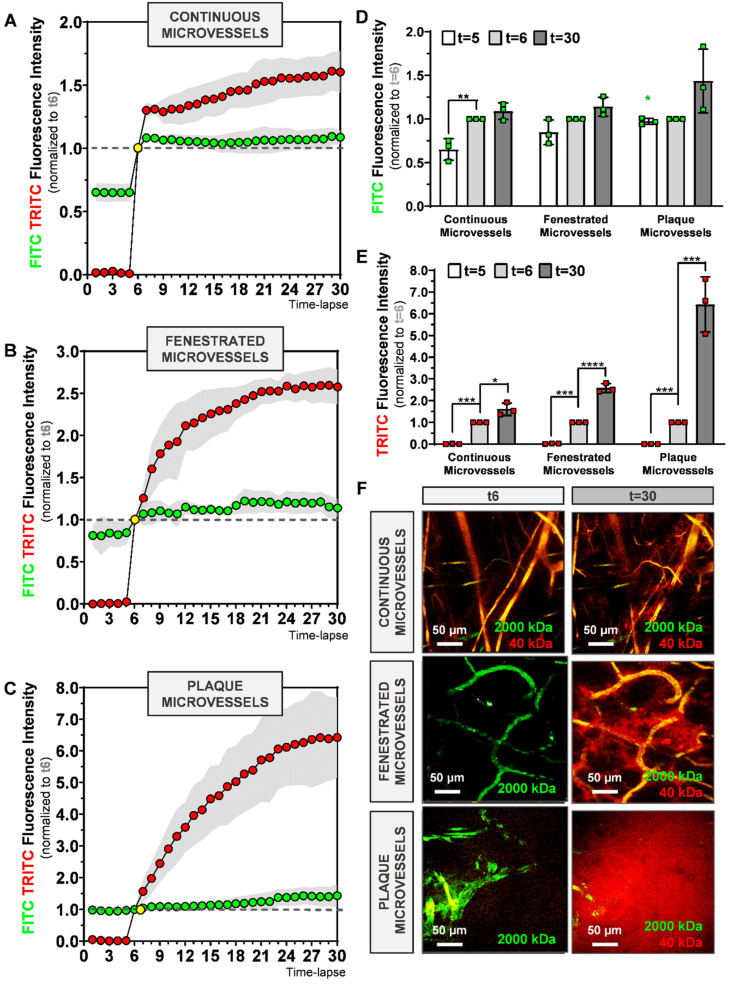
Evaluation of time-lapse imaging of 2000 kDa-FITC and 40 kDa-TRITC RFI outside the microvessel structures of (**A**) continuous, (**B**) fenestrated and (**C**) plaque microvessels. (**D**,**E**) Quantification of FITC and TRITC extravascular RFI at time-lapse 5, 6 and 30. (**F**) Representative max-projection of FITC and TRITC signal in continuous, fenestrated and plaque microvessels at t6 and t30. Data presented as mean ± SD. * *p* ≤ 0.05, ** *p* ≤ 0.001, *** *p* ≤ 0.001, **** *p* ≤ 0.0001 by 1-way-ANOVA; Green star (* *p* ≤ 0.05): FITC RFI at t5 between continuous and plaque microvessels.

Next, we determined if it was possible to visualize dextran extravasation from the vasculature. In the same time-lapse movies as used for Figure 3, we measured the extravascular RFI (normalized to t6) for 2000 kDa-FITC and 40 kDa-TRITC dextran for continuous (Figure 4A), fenestrated (Figure 4B), and plaque (Figure 4C) microvessels.

Residual fluorescence from the 2000 kDa-FITC first injection (*t* ≤ 5) was detected in all groups in the extravascular space as shown in Figure 4A–C. FITC RFI at t5 is different between continuous and plaque microvessels groups (0.652 ± 0.14 vs. 0.973 ± 0.14, *p* = 0.0274, Figure 4D, green star).

After injection of 2000 kDa-FICT and 40 kDa-TRITC dextrans at t5, TRITC extravascular RFI increases significantly between t5 and t6 in all groups, as shown in Figure 3E (*p* < 0.001). However, FITC extravascular RFI (Figure 3D) varies between the groups. While it significantly increases between t5 and t6 in continuous microvessels (*p* = 0.0274), no differences are observed between t5 and t6 (~1) in plaque microvessels (Figure 4C,D).

In the next 24 image recordings, TRITC extravascular RFI increases in all microvessels, as shown in Figure 4A–C. In contrast, FITC extravascular RFI between t6 and t30 does not differ in continuous and fenestrated microvessels, (Figure 3D). However, in plaque microvessels, a trend towards an increase in FITC extravascular RFI is detected at t30 compared to t6, (*p* = 0.0827, Figure 4D).

Importantly, the mean TRITC extravascular RFI at t30 is 1.63 ± 0.29, 63% higher in comparison to t6 (*p* = 0.01) in the continuous microvessels group (Figure 4E). In fenestrated microvessels, TRITC RFI mean at t30 is 2.58 ± 0.21, 158% higher compared to t6 (*p* < 0.0001, Figure 4E). In plaque microvessels, TRITC mean intensity is 6.64 ± 1.27, 564% higher compared to t6 (*p* = 0.0003, Figure 4E). Representative sequences of FITC and TRITC signal in continuous, fenestrated and plaque microvessels, observed in real-time at t0, t6 and t30 are shown in Figure 4F.

Overall, 40 kDa dextran extravasates more than 2000 kDa dextran. 40 kDa dextran continues to extravasate over time, whereas 2000 kDa dextran shows an initial peak in extravasation and then stops.

### 2.4. Comparison Dextrans Extravasation in Continuous, Fenestrated and Plaque Microvessels

**Figure 5:**
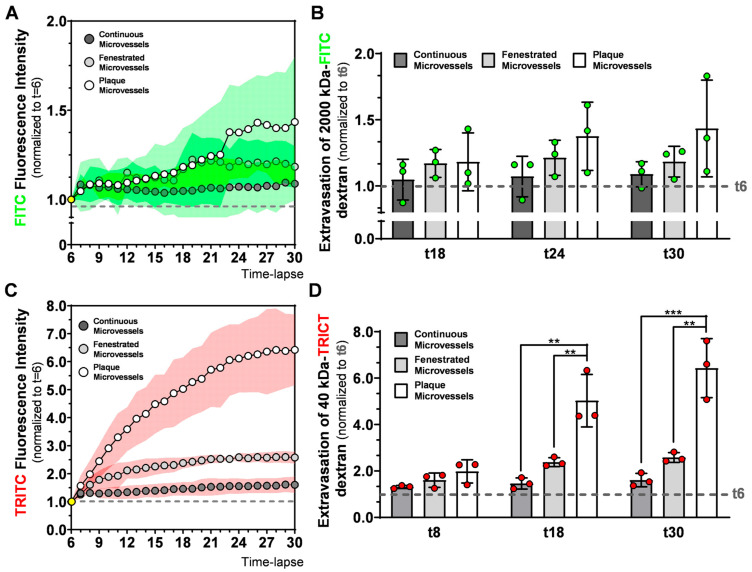
Evaluation of 2000 kDa-FITC and 40 kDa-TRITC dextrans extravasation in continuous, fenestrated and plaque microvessels. Time dependent evolution of (**A**) FITC and (**C**) TRITC extravascular RFI between groups. Comparison of (**B**) 2000 kDa and (**D**) 40 kDa extravasation at different timepoint between groups. Data presented as mean ± SD (SD is expressed as a green (**A**) or red (**C**) cloud). ** *p* ≤ 0.001, *** *p* ≤ 0.001, by 1-way-ANOVA.

To better understand 2000 kDa and 40 kDa dextran extravasation differences between continuous, fenestrated and plaque microvessels, we compared FITC or TRITC fluorescence intensities outside the vessel structures (Figure 5).

FITC extravascular fluorescence intensities show small and non-significant increases over time in all groups as depicted in Figure 5A. Extravasation of 2000 kDa-FITC dextran is comparable between the groups at t18, t24, and t30 as demonstrated in Figure 5B.

The 40 kDa dextran profile displays, strong changes in TRITC extravascular RFI between the groups, which increases in time (Figure 5C). After t6, TRITC intensity increases differently between groups. While in continuous microvessels, TRITC signal increases, reaching a stable value of 1.30 ± 0.10, at t8, in the fenestrated microvessels group, TRITC signal at t8 is already 1.60 ± 0.23 and stabilizes at t18 (2.38 ± 0.19). In plaque microvessels, TRITC RFI raises continuously, with 1.98 ± 0.53 at t8 and 5.03 ± 1.13 at t18. As shown in Figure 5D, extravasation of 40 kDa TRITC-dextran in the plaque microvessels was 3.4-fold higher (*p* = 0.0016) in comparison to continuous microvessels, and 1.8-fold higher (*p* = 0.0072) in comparison to fenestrated capillaries at t18.

At the end of the observation period (t30), TRITC RFI was 1.60 ± 0.29 in continuous microvessels, 2.58 ± 0.11 in fenestrated microvessels and 6.42 ± 1.27 in plaque microvessels (*n* = 3). Extravasation of 40 kDa TRITC-dextran in plaque microvessels was four-fold higher (*p* = 0.0006) compared to continuous microvessels and 2.5-fold higher (*p* = 0.0020) compared to fenestrated capillaries. Thus, continuous, fenestrated, and plaque microvessels are all permeable to 40 kDa size-dextrans but follow different and vessel specific extravasation curves. Importantly, plaque microvessels show the largest permeability.

## 3. Discussion

In this study, we used 2P-IVM to visualize adventitial and intimal plaque microvessels in advanced atherosclerotic lesions in ApoE3*Leiden VGs. In this model, a non-diseased caval vein from a donor mouse is used as an interposition in the carotid artery of a hypercholesterolemic ApoE3*Leiden mouse, within 28 day an atherosclerotic lesion forms with adventitial and intimal plaque microvessels with various forms of maturity [8,9]. We report a 2P-IVM method to evaluate plaque microvessels leakiness in vivo, by comparing the extravasation of 40 kDa dextran in healthy, continuous, and fenestrated, as well as diseased, plaque microvessels. We demonstrated in real-time, that microvessels in advanced atherosclerotic lesions in VGs are pathologically permeable.

By injecting 2000 kDa-FITC dextrans, we were able to observe in vivo microvessels networks throughout the adventitia, media and extending into the intimal layer of the VG lesion. Larger vessels were detected in the adventitia layer, while more narrow vessel structures were detected further deep in the plaque. We here confirm in vivo the abundant presence of intimal microvessels in advance atherosclerotic lesions in the ApoE3*Leiden VG model that was previously shown with histology [8,9]. This is a rare feature seldomly seen in other atherosclerotic mouse models [19,20]. These microvessel networks are characterized by typical vessel features of ongoing angiogenesis, as observed by the irregular microvessel architecture with accentuated turns and branching. Moreover, using 3D and color-depth Z-Stack projections, we demonstrate how vasa vasorum angiogenesis evolves throughout the lesion including the intima.

Fenestrated microvessels (observed in salivary glands) are more permeable than continuous microvessels (located in the ears) due to their increased number of pores, which drives faster molecule diffusion [6,7]. Accordingly, we observed that continuous and fenestrated microvessels follow different 40 kDa dextran extravasation signatures. While in continuous microvessels, extravasation of 40 kDa dextran reached a stable value rapidly in the observation period, extravasation of the 40 kDa dextran in fenestrated microvessels took more time and was more extensive.

Healthy microvessels have a well-organized architecture that acts as a protection barrier but which does let molecules such as nutrients cross. Plaque microvessels, in contrast, have a disorganized structure with a lack of proper pericyte coverage, diminished VE-cadherins junctions, heterogeneous basement membrane and show an unbalance in angiopoietin 1 and 2 expression [9]. This unbalanced architecture leads to dysfunctionality as shown by their co-localization with extravasated erythrocytes and inflammatory cells, in part explained by the increased expression of VCAM-1 and ICAM-1, as previously described by histological analysis [8,9]. By comparing 40 kDa extravasation patterns in healthy microvessels with the plaque microvessels, we demonstrate in real-time, that microvessels in advanced atherosclerotic lesions are pathological permeable. Their 40 kDa-FITC extravasation curves of the plaque microvessels were clearly different and at the end of the observation period, extravascular 40 kDa dextran was four-fold higher compared to continuous microvessels and 2.5-fold higher compared to fenestrated microvessels. This increased permeability can contribute to the extravasation of erythrocytes and leukocytes which drive plaque instability [4].

In the 2000 kDa dextran extravasation patterns in continuous, fenestrated, and plaque microvessels FITC extravascular RFI between t6 and t30 showed a trend towards an increase in the plaque microvessels. FITC extravascular RFI at t30 was similar between the groups. The reason why we were not able to detect different FITC extravascular RFI at t30 might be related to the immature nature of the plaque microvessels. Recent studies have shown that microvessels with an immature structure drive macromolecules accumulation by the enhanced permeability and retention (EPR) effect [21,22].

In this study, we used two injections of 2000 kDa dextran with the same fluorescent dye (FITC), with the first to visualize blood flow and the second to quantify vessel permeability. After the second injection, FITC intravascular RFI increased significantly between t5 and t6, in all groups. However, FITC extravascular RFI between t5 and t6, only increased significantly in continuous microvessels and did not differ in plaque microvessels. Moreover, when we compared FITC extravascular residual fluorescence (from the first injection) at t5 between groups, it was significantly higher in plaque microvessels compared to continuous microvessels.

Therefore, it is possible that 2000 kDa dextran (from the first injection) accumulates in the extravascular space of plaque microvessels due to the EPR effect, thereby decreasing signal differences during quantification of 2000 kDa extravasation in the second injection. In future approaches that aim to quantify extravasation patterns of macromolecules (such as 2000 kDa dextrans) other methods (e.g., dextrans with differently colored dyes) to detect blood flow should be considered.

Nevertheless, this 2P-IVM imaging methodology allows direct imaging of adventitial and intimal microvessels but also the quantification of the permeability of the microvessels by 40 kDa extravasation in a more realistic test environment compared to post-mortem tissue. Moreover, this technique could be easily adapted to further investigated the dynamics of intraplaque angiogenesis and intraplaque hemorrhage. By injecting fluorescent-labelled cells (such as erythrocytes or inflammatory cells), their extravasation, transmigration, and interaction with the endothelium could easily be monitored and quantified in vivo. Therefore, this 2P-IVM method is a promising approach to validate therapeutic angiogenic interventions targeting advance atherosclerosis in preclinical models in real-time.

## 4. Materials and Methods

### 4.1. Animals

All experiments were carried out with approval of the Animal Welfare Committee of the Leiden Medical University Center (28 March 2018; approval number 116002106645-18-096) and in compliance the Directive 2010/63/EU of the European Parliament. Male ApoE3*Leiden mice (*n* = 12), 10–16 weeks old, were fed with a high-cholesterol inducing diet (2.5% cholesterol and 0.05% cholate *w*/*w*, AB diets, Woerden, The Netherlands) during all the experiment. Mice were housed under standard laboratory conditions and received food and water ad libitum.

### 4.2. Vein Graft Surgery

VG surgery consists of the interposition of the caval vein from a donor mouse in the carotid artery of a recipient mouse, as described before [8]. In brief, the recipient mouse was fixed in a supine position, and an incision was made in the neck. The parotid glands were put aside exposing the right carotid artery. Next, the carotid artery was ligated and cut in middle, a cuff was placed at both ends of the arterial segments. Subsequently, the ends were everted over the cuffs and ligated. The vena cava was harvested from the donor mouse and positioned between the carotid artery by sleeving it over the cuffs and tightened with 8/0 sutures. Pulsatile flow through the venous conduit confirms a successful procedure. Finally, the parotid gland is put back in position and the skin is sutured. Within 28 days after the surgery, the VG lesions develop from a few cell layers at the start of the engraftment, to a massive thickened vessel wall [23].

Mice were anesthetized (intraperitoneally) with 5 mg/kg of midazolam (Roche Diagnostics, Basel, Switzerland), 0.5 mg/kg of dexmedetomidine (Orion Corporation, Espoo, Finland) and 0.05 mg/kg of fentanyl (Janssen Pharmaceutical, Beerse, Belgium). After the surgery, the anesthesia was antagonized with 2.5 mg/kg of atipamezole (Orion Corporation,) and 0.5 mg/kg of flumazenil (0.5 mg/kg, Fresenius Kabi, Bad Homburg vor der Höhe, Germany). Then, 0.1 mg/kg of buprenorphine (MSD Animal Health, Boxmeer, The Netherlands) was given for pain relief.

### 4.3. Two-Photon Intravital Microscopy

Four weeks after the surgery (Figure 1A), mice (*n* = 12) were anesthetized and prepared for intravital imaging on a Zeiss LSM 710 NLO upright multiphoton microscope equipped with a Mai Tai Deep See multiphoton laser (690–1040 nm).

Neck and ear regions were shaved, and a catheter was placed in the tail vein for intravenous injections. To image fenestrated microvessels (in the parotid glands) and plaque microvessels (in the VGs), mice were placed in a supine position on an inset located under the microscope (Figure 1B). Parotid glands were extracorporated, and VGs were carefully exposed from the surrounding connective tissue. To image continuous microvessels (in the ear skin), mice were placed in prone position on the inset (Figure 1B). In both positions, temperature was controlled and breathing was monitored. On top of the target tissue, a metal frame was placed. This metal frame was specially designed to allow the use of a water immersion objective (W Plan Apochromat 20×/1.0 DIC M27 75 mm objective) in the different tissues of the mouse body (Figure 1B).

To select areas of interest in the different microvessels types (continuous, fenestrated and plaque), mice were injected with 50 µL of 100 mg/mL FITC-conjugated 2000 kDa dextran (a blood tracer), via the vein catheter (Figure 1B). These areas were imaged by cycles of time-lapse Z-stacks (40 s each) over 20 min by multiphoton excitation at 488 nm (FITC) and 555 nm (TRITC). Emission was collected by two LSM PMTs at 500–558 nm (FITC) and 578–700 nm (TRITC). At the 5th frame of the time-lapse (t5) a mixture of 100 µL of a 100 mg/mL 40 kDa Dextran-TRITC (42874, Sigma-Aldrich, Zwijndrecht, The Netherlands) and 2000 kDa Dextran-FITC (FD2000S, Sigma-Aldrich, Zwijndrecht, The Netherlands) solution was injected to assess vessel permeability in real-time in all the groups (Figure 1C).

To study plaque angiogenesis in particular, a separate group of three mice was used to study the architecture of intimal and adventitial microvessels. Then, 50–150µm depth Z-stacks were acquired by multiphoton excitation at 488 nm, after injection of 50 µL 100 mg/mL FITC-conjugated 2000 kDa dextran, via the tail vein catheter. Directly after imaging, all mice were euthanized by exsanguination.

### 4.4. Data Analysis

#### 4.4.1. Quantification of FITC and TRITC Fluorescence Integrated Density Inside and Outside the Vessel Structures

To quantify FITC and TRITC fluorescence integrated density inside and outside the vessel structures, we converted 2P-IVM time-lapse acquisition files in maximal intensity Z-stack projections (RBG). Based on the literature, 2000 kDa-size dextrans are less prone to extravasate in microvascular structures due to their big size [17]. Therefore, we used the FITC channel to apply a tight automatic threshold and define vessel structures surface, denominated as Vessel Mask. Vessel Mask comprises all pixels inside the vessel value as 1 (defined by the automatic threshold) and all the pixels outside the vessel value as 0, at all the timelapsess. The Outside Mask was created via inversion. Both Vessel Mask and Outside Mask were then multiplied by the FITC and TRITC channel, generating four additional files: FITC pixels inside the vessel, FITC pixels outside the vessel, TRITC pixels inside the vessel, TRITC pixels outside the vessel.

For the all the six files, relative fluorescence intensity (RFI) was calculated by RawIntegrated density function. Since the area between intravascular and extravascular space are different between mice and organs, FITC and TRITC integrated densities were divided by the number of pixels of theVessel Mask or Outside Mask. Subsequently, FITC and TRITC fluorescence integrated densities reflect all the pixel intensities inside or outside the vessel structures. Since 40 kD and 2000 kDa dextran injection was detected intravitally at the 6th frame of the time-lapse (t6), FITC and TRITC fluorescence integrated intensities, intra and extravascular, were normalized to t6 values, and plotted in XY graphs.

#### 4.4.2. Video Processing of FITC and TRITC Fluorescence Time-Lapse Series Acquired by 2P-IVM

To evaluate 40 kDa and 2000 kDa dextran extravasation patterns, we generated video time-lapse series of maximal FITC and TRITC fluorescence Z-stack projections. 2000 kDa dextran is visualized in the green channel and 40 kDa dextran is visualized in the red channel.

Plaque microvessels in advanced atherosclerotic VG lesions were processed by maximal FITC fluorescence Z-stack projections. Moreover, we generated 3D stack projections using Imaris 3D rendering software (Imaris, Zurich, Switzerland). In both renderings 2000 kDa-FITC dextran is visualized in the green channel.

To distinguish between adventitial and intimal microvessels, we used the temporal color-coding plugin in ImageJ FIJI that used a color LUT based on Z-depth and projects this in a maximum projection image.

### 4.5. Statistical Analysis

Results are shown as mean ± standard deviation error (SD). One-way ANOVA was used to compare differences between groups. Differences were considered significant when *p* * ≤ 0.05, *p* ** ≤ 0.01, *p* *** ≤ 0.001 or *p* **** ≤ 0.001.

## Abbreviations

2P-IVM	Two-photon intravital microscopy
RFI	Relative fluorescence intensity
